# CD90-positive stromal cells associate with inflammatory and fibrotic changes in modic changes

**DOI:** 10.1016/j.ocarto.2022.100287

**Published:** 2022-06-22

**Authors:** Stefan Dudli, Agnieszka Karol, Luca Giudici, Irina Heggli, Christoph J Laux, Jose M Spirig, Florian Wanivenhaus, Michael Betz, Christoph Germann, Nadja Farshad-Amacker, Florian Brunner, Oliver Distler, Mazda Farshad

**Affiliations:** a Center of Experimental Rheumatology, University Hospital Zurich, University of Zurich, Switzerland; b Department of Molecular Mechanisms of Disease, University of Zurich, Switzerland; c Institute of Pathology and Molecular Pathology, University Hospital Zurich, University of Zurich, Switzerland; d Department of Orthopeadics, Balgrist University Hospital, University of Zurich, Switzerland; e Department of Radiology, Balgrist University Hospital, University of Zurich, Switzerland; f Department of Physical Medicine and Rheumatology, Balgrist University Hospital, University of Zurich, Switzerland

**Keywords:** Low back pain, Modic changes, Bone marrow oedema, Fibrosis, Inflammation

## Abstract

**Objective:**

Modic changes (MC) are vertebral bone marrow lesions seen on magnetic resonance images, that associate with disc degeneration and low back pain (LBP). Few studies described MC histopathology qualitatively based on a few patient samples. CD90-positive bone marrow stromal cells were shown to be pro-fibrotic in MC. We aimed to provide the first semi-quantitative histomorphometric analysis of MC bone marrow. We hypothesized a role of CD90-positive cells in MC pathomechanisms.

**Design:**

Human biopsies from Modic type 1 changes (MC1, n ​= ​8), Modic type 2 changes (MC2, n ​= ​6), and control biopsies (MC0, n ​= ​8) from adjacent vertebrae were obtained from 14 LBP patients during lumbar spinal fusion. Biopsies were processed for histology/immunohistochemistry. Inflammatory changes (oedema, inflammatory infiltrates), fibrotic changes (connective tissue, type I and III collagen, fibronectin, α-smooth muscle actin), and amount of bone marrow stromal cells (CD90, CD105) were scored. Scores for MC0, MC1, and MC2 were compared with non-parametric tests. Pairwise correlations, hierarchical clustering, and principal component analysis of histological readouts were calculated to identify most important histomorphometric MC characteristics.

**Results:**

Compared to MC0, MC1 had more connective tissue, oedema, inflammatory infiltrates, and CD90^+^ cells. MC2 compared to MC0 had more oedema and CD90^+^ cells. Scores of CD90 correlated and clustered with inflammatory and fibrotic changes. Amount of connective tissue correlated with LBP.

**Conclusion:**

Accumulation of CD90^+^ cells is a major characteristic of MC in patients undergoing lumbar spinal fusion and associates with inflammatory and fibrotic changes. Therefore, CD90^+^ cells may play an important role in the inflammatory-fibrotic pathomechanisms of MC.

## 1 Introduction

Modic changes (MC) are vertebral bone marrow lesions adjacent to degenerated intervertebral discs [1]. MC are diagnosed based on T1-weighted (T1w) and T2-weighted (T2w) magnetic resonance images (MRI). Three interconvertible types of MC exist: MC type 1 (MC1) is hypointense on T1w and hyperintense on T2w, MC type 2 (MC2) is hyperintense on T1w and T2w images, and MC type 3 (MC3) is hypointense on T1w and T2w images. Mixed types, mainly MC1/MC2 and MC2/MC3 also exist [2]. MC, and in particular MC1, strongly associate with chronic LBP, are highly specific for discogenic low back pain (LBP), correlate with endplate damage, and are a risk factor for poor 14-month outcome of conservative treatment [[3], [4], [5], [6], [7]]. While MC lesions only affecting the endplates often resolve, lesions affecting substantial marrow volumes rarely resolve [8]. Despite MC prevalence's of 15–65% in chronic LBP patients and relevant and strong pain association [5,9,10], the histopathology of MC remains poorly understood. The only published histological data from clinical MC biopsies are qualitative descriptions [1,[11], [12], [13]]. They reported fibrovascular granulation tissue with lymphocytic infiltrates and high bone turnover in MC1, and fatty marrow with reduced bone remodelling in MC2. Analysis of bone marrow cells revealed pro-inflammatory and pro-fibrotic changes in MC1 and MC2 [14] and that Thy-1 (CD90) positive bone marrow stromal cells (BMSCs) are pro-fibrotic in MC1 [15]. However, quantitative histomorphometric data are missing. This would reveal the main characteristics of MC and provide a solid basis for pathomechanistic studies. Therefore, we performed the first semi-quantitative analysis of human MC bone marrow with focus on inflammatory and fibrotic changes. We specifically investigated the presence of CD90^+^ BMSC and hypothesized that CD90^+^ BMSC associate with fibrotic and inflammatory changes in MC.

## 2 Materials and methods

### 2.1 Patients

Ethical approval to collect and analyse bone marrow biopsies and patient data from patients undergoing transforaminal lumbar interbody fusion at the Balgrist University Hospital was obtained from the Ethics commission of the Canton of Zurich, Switzerland (BASF 2017–00761). Each patient gave informed consent to participate in this study. The study was conducted in accordance with the Helsinki Declaration of 1975, as revised in 2000. Indication for surgery and painful spinal level were identified with clinical examinations and with reviewing radiologic data. Indications for surgery were degenerative disorders needing spinal fusion such as foraminal stenosis due to segmental degeneration, isthmic spondylolisthesis, degenerative spondylolisthesis, and degenerative scoliosis. All patients had also radicular leg pain due to nerve compression and none had isolated low back pain. Exclusion criteria were infectious diseases, tumours, prior instrumented back surgery, rheumatic markers (HLA, autoantibodies) if reported, juvenile scoliosis. Patients were identified pre-operatively based on T1-weighted and T2-weighted lumbar MRI. Scanner information is given in Supplementary Data 1. Patients reported preoperative back and leg pain on a 10-point visual analogue score (VAS back and VAS leg, respectively) and filled out the Oswestry-Disability-Index questionnaire (ODI, version 2.1). Symptom duration was recorded.

**Fig. 1:**
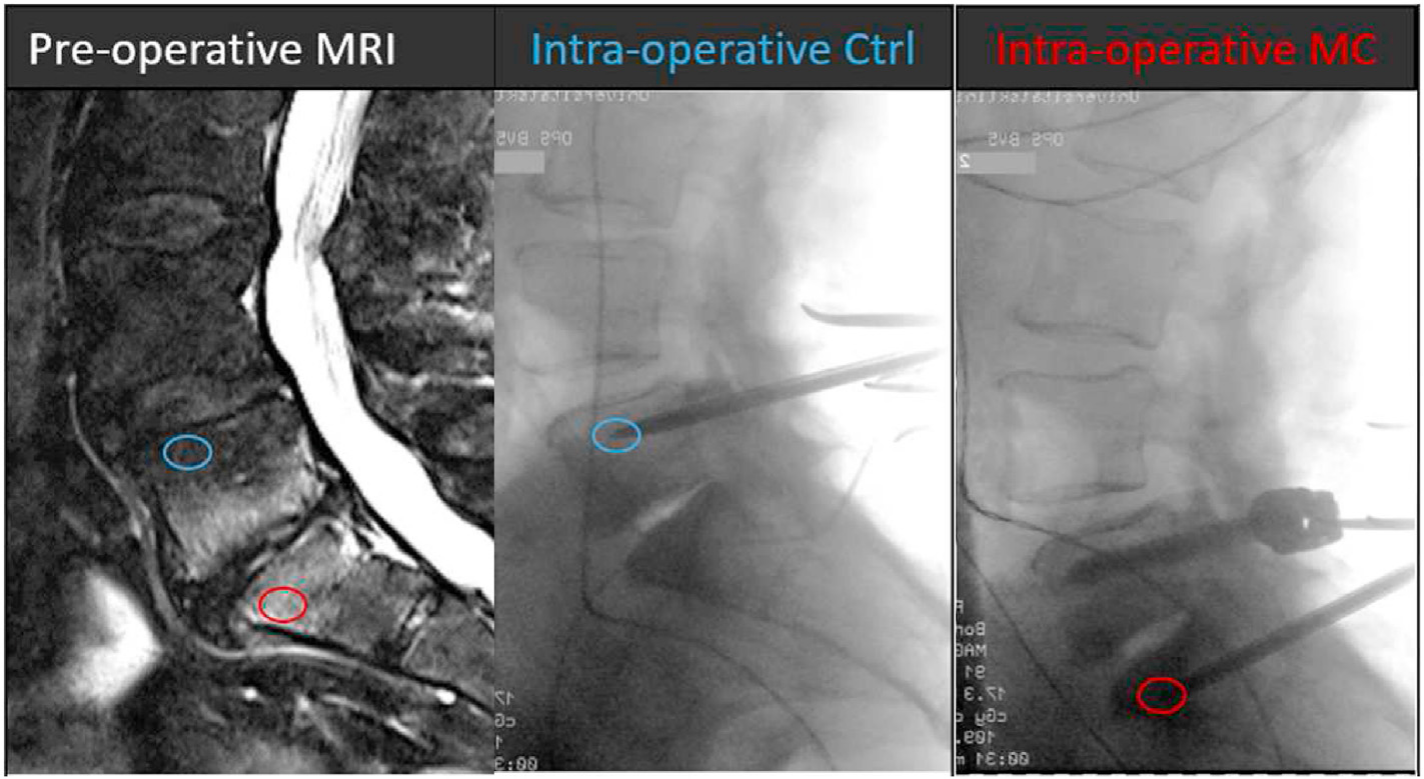
Intra-operative sampling of vertebral bone marrow biopsies. Patients with lumbar MC were identified pre-operatively on MRI (left). Biopsies were taken intra-operatively with Jamishidi needles (middle and right). Intra-operative X-ray was used to confirm proper needle position. A control (blue circle) and a MC (red circle) biopsy were taken.

Spondylodesis requires the insertion of pedicle screws into the vertebral bodies. Using the same trajectories, bone marrow biopsies can be taken before screw insertion. Proper positioning of the biopsy needle was key. Therefore, we only included patients with large MC lesions where we were sure that the needle came to lie within the MC lesion. Intraoperatively, we confirmed biopsy site with X-ray. Post-operatively, proper needle position was confirmed by comparing intra-operative X-ray with pre-operative MRI (Fig. 1). A total of 22 biopsies were taken from fourteen patients undergoing lumbar spondylodesis at the Balgrist University Hospital between February 2019 and September 2020. If possible, a MC and control (MC0) biopsy were taken from the same patient. From seven patients, only one biopsy was taken.

### 2.2 Radiological readout

Two experienced board-certified musculoskeletal subspecialized radiologists with over 8 years of experience independently classified MC type (MC0, MC1, MC2, MC3). Disc degeneration was graded (Pfirmann grade 0–5) and endplate degeneration scored (EPS) (0–6) [16,17]. Mean values of the two read-outs were taken.

### 2.3 Histology and immunohistochemistry

Biopsies were transferred immediately in the operating room into 4% buffered formalin solution (Sigma Aldrich, Buchs, Switzerland). The next day, biopsies were decalcified in 12.5% ethylenediaminetetraacetic acid (Sigma Aldrich, Buchs, Switzerland) for four days. Biopsies were dehydrated in increasing concentrations of ethanol (Sigma Aldrich, Buchs, Switzerland), embedded in paraffin, and cut into 5 ​μm sections. Sections were stained with hematoxylin/eosin (HE) (Sigma Aldrich, Buchs, Switzerland) and Masson trichrome (MT) (Sigma Aldrich, Buchs, Switzerland). Tissues were classified as homogeneous or heterogeneous by comparing several regions of interest. HE slides were scored for cellularity, inflammatory infiltrates, oedema (as an excess of interstitial liquid content), and adiponecrosis. MT slides were evaluated for deposition of fibrotic tissue. With immunohistochemistry, positivity of CD90 and endoglin (CD105) was scored as measure for the presence of BMSC. Alpha smooth muscle actin (αSMA) was scored as measure for myofibroblasts or activated BMSC, type I collagen (COL1), type III collagen (COL3), and cellular fibronectin (FN) were scored as measure for fibrosis. For antibody information please see Supplementary Data 2. Scoring scheme for all histology and immunohistochemistry readouts can be found in Supplementary Data 3. Slides were scored by two experienced pathologists. Mean values of the two readers were taken for further analysis. Histological evaluation was performed in focus of diagnostic findings (tissue reaction/alteration), their localization within a tissue and the severity grade. A semiquantitative grading system was used to define a severity of gradable changes (1 ​= ​minimal, 2 ​= ​mild, 3 ​= ​moderate, 4 ​= ​marked), as well as their distribution within the examined tissue (1 ​= ​focal, 2 ​= ​multifocal, 3 ​= ​coalescing and 4 ​= ​diffuse). The two scores were added (A ​+ ​B) as measure for total tissue alteration. Slides were scanned with a Zeiss Axio Scan. Z1 slide scanner (Carl Zeiss Microscopy GmbH, Jena, Germany) at 20× magnification or with a Leica DMR system (Leica Biosystems GmbH, Nussloch, Germany). Images were viewed with Zeiss ZEN Blue v3.1 (Carl Zeiss Microscopy GmbH, Jena, Germany) or Imagic Image Managements System Client (Imagic bildverarbeitungs AG, Glattbrugg, Switzerland) and representative areas were captured.

### 2.4 Statistics

Statistical analysis was done in R version 3.6.2 (R Core Team). Radiologic and histologic readouts were compared between MC types (MC0, MC1, MC2) with Kruskal Wallis tests. Fisher exact tests were used to detect significant proportion changes of categorical variables (sex, smoker, tissue homogeneity) among MC types. Holm correction was used to correct pairwise p-values for MC0, MC1, and MC2. For binomial data (i.e. homogeneous/heterogeneous tissue composition) generalized linear models were calculated to test for relationships with quantitative data. Inter-class correlation coefficients were calculated for histologic readouts and reported in Supplementary Data 3. Radiologic and histologic readouts were correlated using Kendall's tau correlation. Correlations were classified as poor (<0.40), moderate (0.40–0.59), strong (0.60–0.74), and very strong (0.75–1.00). Principal component analysis (PCA) of scaled histological readouts was calculated to identify the most important histomorphometric MC characteristics. Scaling was done with the function prcomp () in order that histological readouts had unit variance. The first two principal components were considered based on an elbow plot. Histological data were clustered using hierarchical clustering of Euclidean distance matrices based on scaled histological readout scores to identify most similar parameters. In order to stratify specimens into relatively homogeneous subgroups, patient's histological readouts were clustered using k-means clustering based on a scaled distance matrix. Silhouette plots were used to identify the optimal number of clusters (Supplementary Data 4).

## 3 Results

There were no differences in patient demographics between groups (Table 1). Connective tissue in bone marrow measured as Masson-trichrome staining intensity and distribution correlated with back pain (VAS back) (τ ​= ​0.51, p ​= ​0.033). All other histologic and radiologic readouts did not correlate with demographic parameters (height, weight, smoker, VAS back, VAS leg, ODI, age, sex, symptom duration).

**Table 1:** **Patient demographics.** Data are shown as mean values ​± ​standard deviation, except for male sex and smokers, where number of individuals are reported. Data in parentheses are percentages. VAS.back and VAS.leg: Visual Analogue Score for back and leg pain, respectively. ODI: Oswestry Disability Index. Symptoms: duration of low back pain symptoms. MC0: no Modic change. MC1: Modic type 1 change. MC2: Modic type 2 change.

	age (yr)	male (%)	height (cm)	weight (kg)	smoker (%)	VAS.back (0–10)	VAS.leg (0–10)	ODI (0–100%)	symptoms (yr)
Patients (n ​= ​14)									
mean ​± ​sd	62.7 ​± ​11.8		171.2 ​± ​11.2	86.1 ​± ​18.2		5.9 ​± ​2.3	5.5 ​± ​3.0	38.5 ​± ​16.0	4.4 ​± ​5.1
percentage		10 (71.4)			9 (64.3)				
Biopsies (n ​= ​22)									
MC0 (n ​= ​8)	63.0 ​± ​12.3	5 (62.5)	166.9 ​± ​10.0	79.0 ​± ​15.3	5 (62.5)	5.4 ​± ​2.6	5.6 ​± ​2.7	38.1 ​± ​18.0	4.2 ​± ​5.6
MC1 (n ​= ​8)	58.0 ​± ​9.7	5 (62.5)	170.0 ​± ​10.8	83.8 ​± ​20.1	7 (87.5)	6.6 ​± ​1.4	5.3 ​± ​2.6	40.0 ​± ​15.3	2.9 ​± ​3.2
MC2 (n ​= ​6)	64.2 ​± ​11.0	5 (83.3)	174.7 ​± ​13.9	90.6 ​± ​22.7	4 (66.7)	5.8 ​± ​1.9	7.2 ​± ​2.1	40.8 ​± ​10.3	5.6 ​± ​5.2
*p-value*	*0.58*	*0.724*	*0.517*	*0.593*	*0.59*	*0.626*	*0.476*	*0.959*	*0.619*

### 3.1 Fibrotic and inflammatory changes in MC bone marrow

**Fig. 2:**
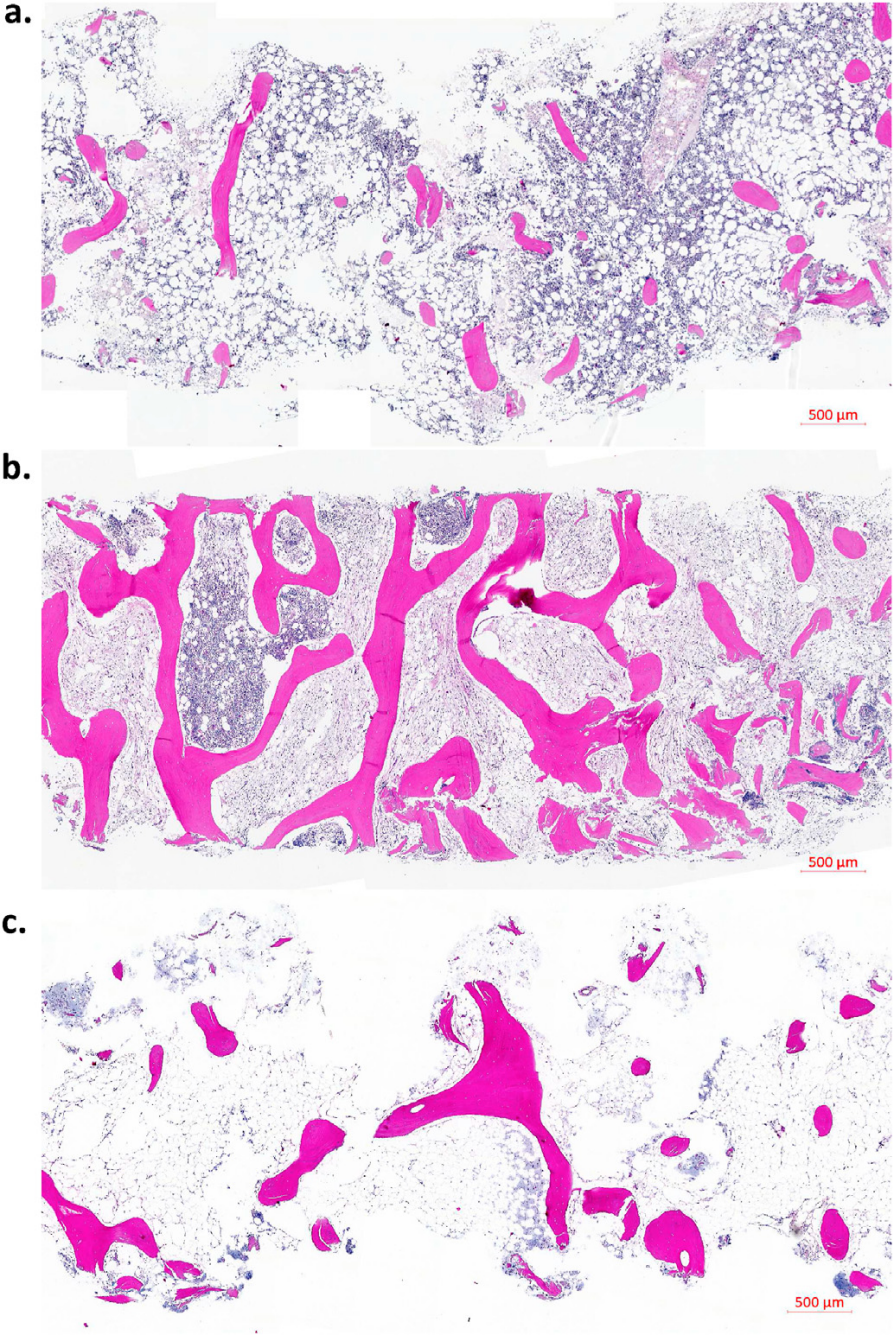
Comparison of vertebral biopsies from three different patients: (a) MC0 control, (b) MC1, and (c) MC2. Hematoxylin-Eosin stain. Magnification 20×.

**Fig. 3:**
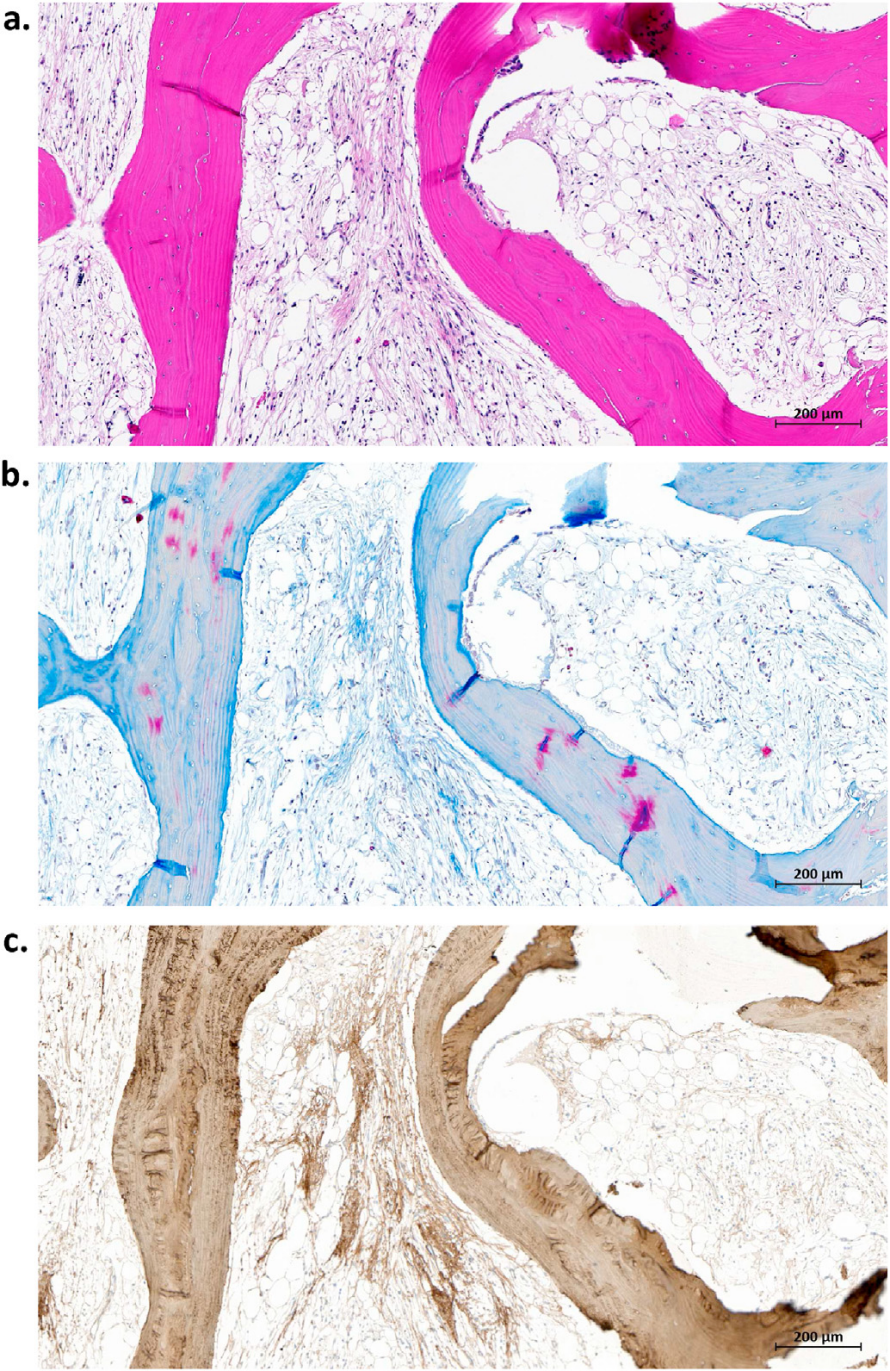
Fibrosis in MC1. Same region of interest stained with (a) Hematoxylin-Eosin, (b) Masson-Trichrome, and (c) type I collagen (immunostain). Magnification: 20×.

**Fig. 4:**
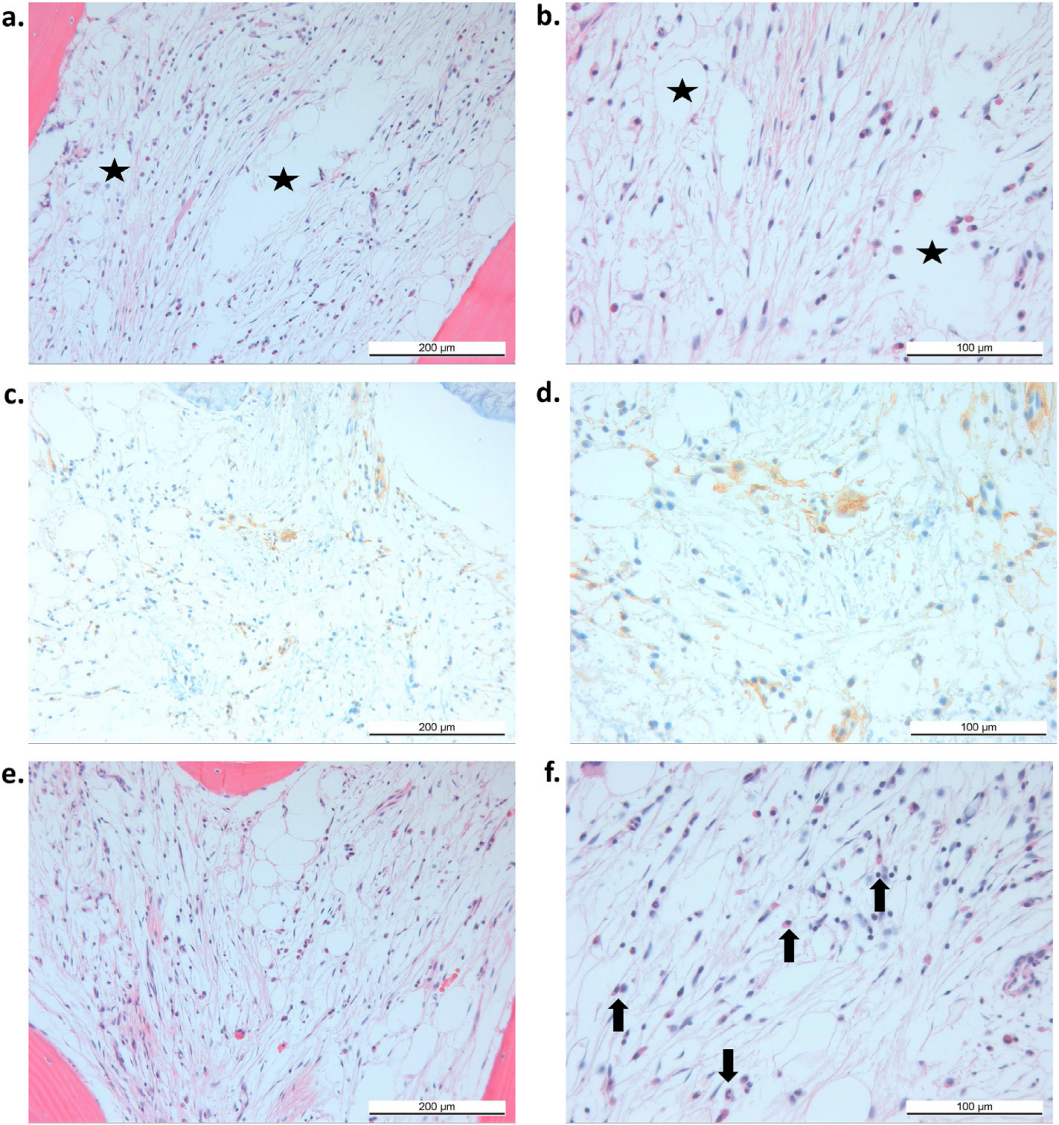
Oedema, CD90^+^ cells, and inflammatory infiltrates in MC1. (a–b) Loosened connective tissue (hollow spaces, asterisk) in the intertrabecular space (oedema). (c–d) CD90 immunopositive cells (brown DAB substrate, membrane signal). (e–f) Inflammatory infiltrates (black arrow) consisting of lymphocytes, plasma cells, and to a lesser extent of neutrophil granulocytes. Stains: Hematoxylin-Eosin (a, b, e, f), CD90 immunohistochemical stain (c, d). Magnifications: 100× (a, c, e), 200x (b, d, f).

There was an increase of connective tissue (MT) in MC1 (Fig. 2, Fig. 3), and more CD90^+^ cells and more oedema in MC1 and MC2 (Fig. 4) (Table 2). While none of the control biopsies showed signs of oedema, in MC1 (5 of 8 (62.5%), p ​= ​0.051) and MC2 biopsies (4 of 6 (66.6%), p ​= ​0.045) oedema was detected. Most control biopsies showed no CD90-positive cells (1 of 8, 12.5%), in contrast most MC1 biopsies (7 of 8, 87.5%, p ​= ​0.030) had CD90-positive cells. In MC1, inflammatory infiltrates tended to be more frequent. Most common infiltrates in MC were plasma cells and lymphocytes (Table 3). Granulocytic infiltrates were found in two (25%) MC1 biopsies, but not in MC2 and MC0.

**Table 2:** **Radiologic and histological results**. Disc degeneration (DD) and endplate scores (EPC) were graded on MRI at vertebral levels, where biopsies were taken. Control (MC0), Modic type (MC1), and Modic type 2 (MC2) were compared with Kruskal Wallis tests (Kruskal), except for tissue homogeneity/heterogeneity, where a Fisher exact test was performed. *P*-values of pairwise comparisons were adjusted according to Holm's method. Significant values are highlighted in bold. Power of post-hoc power-analysis is indicated in the last column. MT: Masson-trichrome. αSMA: alpha smooth muscle actin. COL1: type I collagen. COL3: type III collagen. FN: cellular fibronectin. CD105: endoglin. CD90: Thy-1.

				p-value			
	MC0 (n ​= ​8)	MC1 (n ​= ​8)	MC2 (n ​= ​6)	Kruskal	MC0-MC1	MC0-MC2	MC1-MC2
DD	3.69 ​± ​0.8	4.94 ​± ​0.18	4.67 ​± ​0.41	**0.001**	< ​**0.001**	< ​**0.001**	0.081
EPS	2.06 ​± ​0.62	5.75 ​± ​0.38	4.67 ​± ​1.21	**0.000**	< ​**0.001**	< ​**0.001**	**0.045**
cellularity	1.56 ​± ​0.68	1.13 ​± ​0.74	1.5 ​± ​0.77	0.492	0.899	0.953	0.899
homo/hetero	6/2	1/7	2/4	**0.042**	0.122	1.000	0.182
inflammation	0.38 ​± ​0.69	1.88 ​± ​1.69	0.5 ​± ​0.77	0.081	0.105	0.807	0.158
oedema	0 ​± ​0	1.13 ​± ​0.99	0.83 ​± ​0.75	**0.019**	**0.015**	**0.044**	0.660
MT	0.44 ​± ​0.82	2.19 ​± ​1.53	1.58 ​± ​1.11	**0.034**	**0.027**	0.126	0.480
αSMA	0.63 ​± ​0.44	1 ​± ​0.71	1.08 ​± ​0.2	0.166	0.479	0.199	0.479
COL1	2.63 ​± ​1.19	3.69 ​± ​1.75	3.25 ​± ​0.52	0.310	0.461	0.607	0.754
COL3	5.88 ​± ​1.03	5.94 ​± ​1.12	5.42 ​± ​1.16	0.445	0.774	0.710	0.710
FN	3.63 ​± ​1.69	4.25 ​± ​2.55	4.17 ​± ​0.75	0.712	1.000	1.000	1.000
CD105	1.69 ​± ​1.33	3.19 ​± ​2.36	1.63 ​± ​1.22	0.266	0.511	0.904	0.511
CD90	0.13 ​± ​0.35	3.34 ​± ​2.33	1.88 ​± ​1.7	**0.005**	**0.001**	0.052	0.130

**Table 3:** Number of biopsies with inflammatory cell infiltrates in bone marrow with Modic type 1 changes (MC1), Modic type 2 changes (MC2), and control (MC0) bone marrow. ‘None’ indicates number of biopsies without any inflammatory infiltrates.

	MC0 (n ​= ​8)	MC1 (n ​= ​8)	MC2 (n ​= ​6)
Plasma cells	2 (25%)	4 (50%)	1 (16.7%)
lymphocytes	2 (25%)	4 (50%)	2 (33.3%)
macrophages	2 (25%)	3 (37.5%)	2 (33.3%)
granulocytes	0 (0%)	2 (25%)	0 (0%)

### 3.2 Parameter correlations

**Fig. 5:**
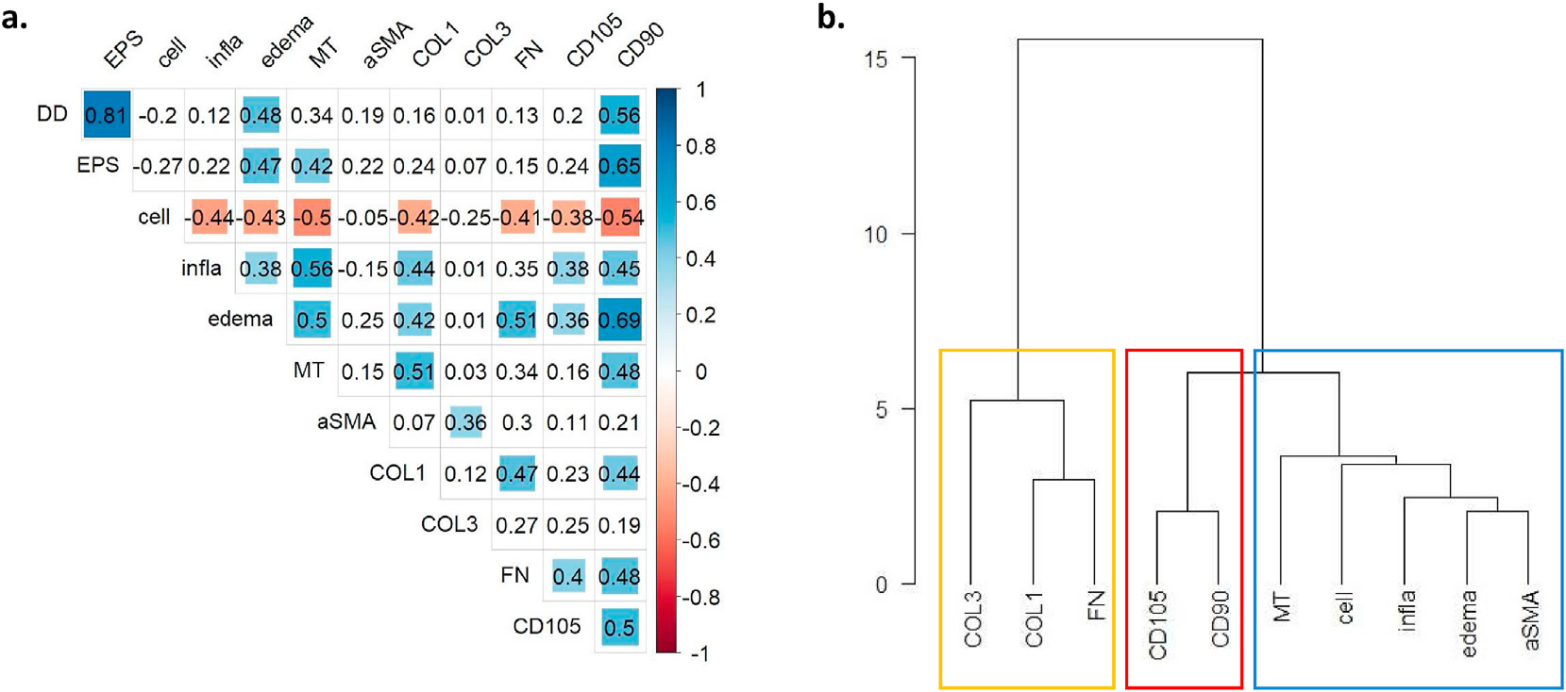
**(a)** Correlation matrix of radiologic and histologic readouts. Kendall's tau correlation coefficients are indicated. Coloured squares indicate significant correlations (p ​< ​0.05). Sizes of squares are proportional to the absolute value of tau. Blue colour: tau>0, red colour: tau<0. **(b)** Hierarchical clustering of histological readouts. Blue rectangle indicates inflammatory cluster, red rectangle bone marrow stromal cells. EPS: endplate score. Cell: cellularity. Infla: inflammatory infiltrates. MT: Masson-trichrome. αSMA: alpha smooth muscle actin. COL1: type I collagen. COL3: type III collagen. FN: cellular fibronectin. CD105: endoglin. CD90: Thy-1.

CD90-positive BMSCs correlated with CD105, with inflammatory (inflammatory infiltrates, oedema) and fibrotic (MT, COL1, FN) bone marrow changes, and with degenerative disc changes (DD, EPS) (Fig. 5a). Degenerative disc changes further correlated with bone marrow oedema and connective tissue content (MT). Bone marrow cellularity was negatively correlated with all other readouts. Hierarchical clustering identified two major clusters (Fig. 5b): a cluster with measures for bone marrow fibrosis (yellow rectangle) and a second cluster consisting of BMSC markers (red rectangle) and inflammatory measure (blue). Marker for stromal cells were closer related to inflammatory markers than fibrotic markers.

### 3.3 Inconsistent tissue changes in MC

**Fig. 6:**
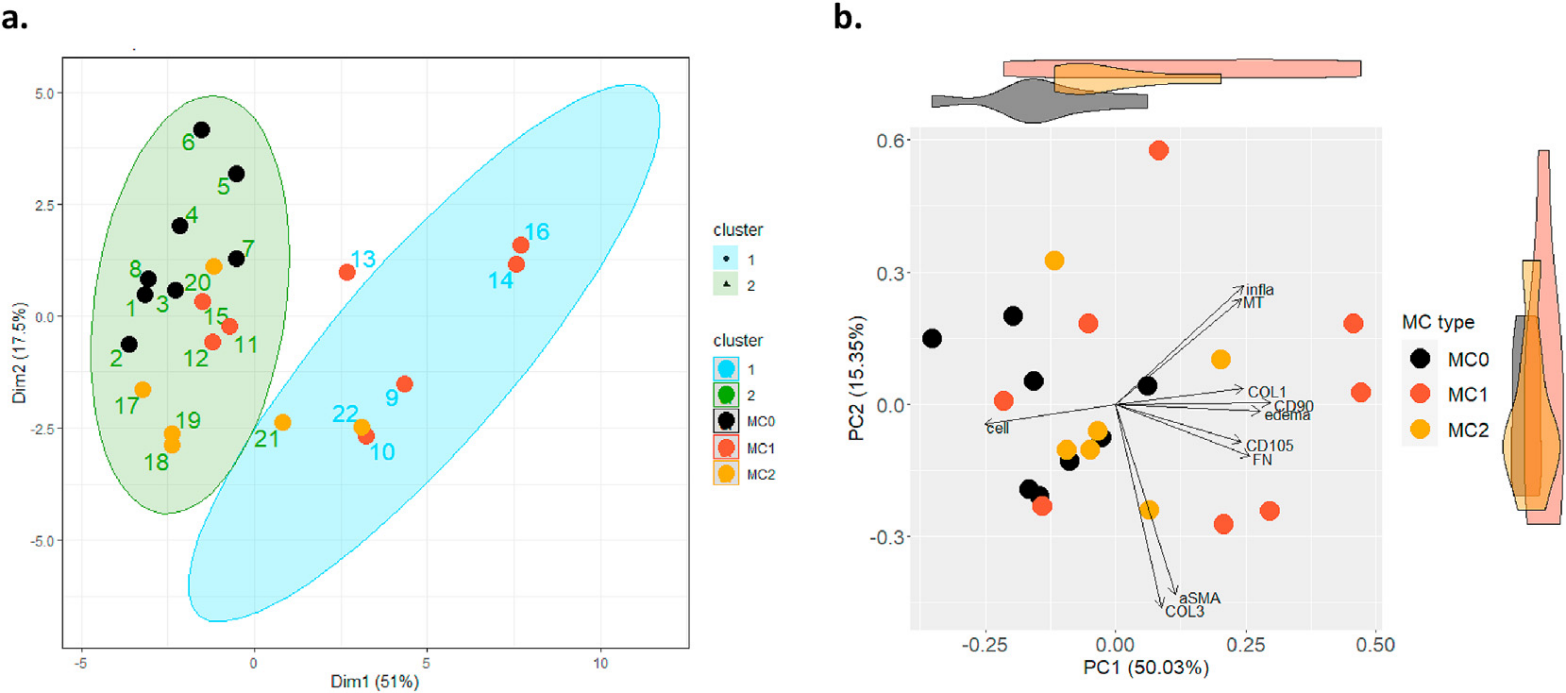
Clustering of biopsies and principal component analysis of histological readouts. (a) K-means clustering of histological readouts identified two clusters. Cluster 2 contains all MC0 biopsies, 4 of 6 MC2 biopsies and 3 of 8 MC1 biopsies. Cluster 1 only contained MC1 or MC2 biopsies. (b) Principal component analysis of histological readouts of biopsies from control bone marrow (MC0), Modic type 1 change (MC1) and Modic type 2 change (MC2) bone marrow. Principal component 1 (PC1) and principal component 2 (PC2) explained over 65% of the variance. Loadings of the histological readouts are presented as arrows. Violin plots on top and the right show distribution of the biopsies on PC1 and PC2.

With k-means clustering and PCA of histological readouts, we identified the most important characteristics of MC and showed an increased variance in tissue composition in MC1 compared to control. K-means clustering of histological readouts identified two clusters (Fig. 6a). One cluster contained all MC0 biopsies, 5 of 6 MC2 biopsies and 3 of 8 MC1 biopsies (cluster 2). The other cluster only contained MC biopsies (cluster 1). We compared MC biopsies (MC1 and MC2 pooled) between cluster 1 and cluster 2 and found no difference in demographic and radiologic measures. Differences were found for CD90, oedema, inflammatory infiltrates, fibronectin, connective tissue, and cellularity (Supplementary Data 5). With PCA, we confirmed that these parameters account for most variance of the first two principal components (Fig. 6b). The first principal component (PC1) explained over 50% of the variance and contrasts bone marrow cellularity with CD90, CD105, oedema, COL1, and FN. The single most important contributor to PC1 was CD90 (16%), followed by oedema (13.9%). The second principal component (PC2) explained another 15.4% of the variance and contrasts inflammatory infiltrates and MT with αSMA and type III collagen, with type III collagen (38.4%) and fibronectin (33.4%) contributing most. MC1 showed largest variation on both, PC1 and PC2 and fully overlapped with MC2. Control biopsies (MC0) strongly overlapped with MC1 and MC2, indicating that some MC1 and MC2 samples were not different from MC0 with respect to PC1 and PC2.

## 4 Discussion

Fibrosis, granulation tissue, oedema, lymphocytic infiltrates, increased adipocyte frequency, and adiponecrosis have anecdotally been reported from the investigation of a few MC biopsies [1,11,12]. Here, we performed the first semi-quantitative analysis of MC bone marrow from patients with chronic LBP. We found in average strong fibrotic and inflammatory changes in MC1 and moderate changes in MC2 bone marrow. These changes correlated with the number of CD90^+^ BMSCs suggesting a role of CD90^+^ BMSC in the pathomechanisms of MC. While this does not imply any causality, it substantiates their involvement in fibrotic-inflammatory mechanisms in MC and provides a basis for further mechanistic studies.

Stromal cells play important mechanistic roles in joint diseases. In osteoarthritic bone marrow lesions, BMSCs are more frequent and show age-related changes [18,19]. In the synovium of OA joints, the number of synovial CD90^+^ fibroblasts correlates with disease severity and in rheumatoid arthritis, CD90^+^ fibroblasts denote an inflammatory fibroblast subset that associate with the amount of joint immune cell infiltrates [20,21]. In primary myelofibrosis, BMSCs drive fibrosis of the bone marrow [22]. In MC1, a recent study showed that CD90^+^ BMSC are more frequent and have a pro-fibrotic phenotype without increasing αSMA expression [15]. Here, we confirmed histologically, that CD90^+^ BMSCs co-occur with fibrosis. Fibrosis in MC seem to be clinically relevant, because fibrotic serum biomarkers correlate with presence of MC [23], and because we showed here that the amount of connective tissue in the bone marrow correlates with LBP intensity.

Fibrosis and inflammation in MC are linked to fibrotic and inflammatory changes in the adjacent disc [14], yet the mechanisms of the disc-marrow cross-talk are unknown. In this study, we found that DD does not correlate with fibrotic markers in the bone marrow but with CD90^+^ BMSC and bone marrow oedema. This indicates that fibrotic marrow changes in MC do not progress with DD but that progressive DD is linked to expansion of CD90^+^ BMSC and oedema. With PCA, we found that an increase in CD90^+^ cells is a main characteristic of MC bone marrow. The strong correlation of CD90 with oedema and the clustering of CD90/CD105 with inflammatory markers suggest that BMSCs in MC are markers of local inflammatory mechanisms. Since MC BMSC do not secret more pro-inflammatory cytokines than intra-patient control BMSC [15], the link to inflammation is indirect, potentially without causality. Repeated and accumulating structural damage of the endplate may cause fibrotic changes by triggering healing attempts of BMSC with increased proliferation and with a fibrotic phenotype shift. In addition, endplate damages can also cause inflammation directly through release of lactate dehydrogenase (LDH) from damaged cells [24,25], or indirectly through pro-inflammatory changes in the disc [26,27]. Therefore, different mechanisms may lead to the co-occurrence of CD90^+^ BMSCs with fibrosis and inflammation. Direct attraction and activation of immune cells by MC BMSC is unlikely based on current knowledge of MC BMSCs.

The sequence of pathological events leading to fibrosis and inflammation in MC is unknown. Conversions between MC1 and MC2 occur, and mixed-type MC1/2 exist. Therefore, results of histological studies may depend on the history and time point of investigation and of the precise location of the biopsy [8].

We aimed to identify the most important characteristics of MC biopsies with PCA. We found that CD90 is a major characteristic of MC together with inflammatory and fibrotic changes. These parameters were contrasted only by reduced cellularity. This indicates that an increase in CD90^+^ BMSC co-occurs with inflammatory and fibrotic changes at the expense of hematopoietic cellularity. PCA also revealed a large variation among tissue composition of MC1 biopsies. While some MC1 are different from MC0 with respect to PC1 and PC2, others are identical to MC0. Changes in MC2 were between MC1 and MC0. The biopsies are small samples from a larger and possibly heterogeneous lesion and hence the histopathologic findings may not be representative for the entire lesions. The large variation of histopathological findings in MC1 may reflect the heterogeneity across a MC1 lesion. Alternatively, the heterogeneity in MC1 may indicate subtypes of MC1 documenting different histories of the MC lesion (e.g. initial vs. recurrent) or reflecting different aetiologies, i.e. autoinflammation against disc material vs. subclinical infection of the disc with *Cutibacterium acnes* [8,[28], [29], [30], [31], [32]]. In this study, we did not analyse the adjacent discs for *C. acnes* and hence cannot conclude if heterogeneity in MC1 histomorphometry relates to different etiological factors.

A limitation of this study is the limited sample size. This is the first quantitative histological study on Modic changes and no data were available prior to the study that allowed to calculate sample size. The here provided quantitative data enables to design larger and well powered studies investigating specific mechanisms of Modic changes. Taking bone marrow biopsies from MC patients requires lesions that are large enough to place the bone marrow needle with high certainty into the MC lesion. We only took biopsies from large lesions where we were sure that the needle came to lie in MC lesions. This excluded many patients. Large biological variation in the few analysed samples increased type II errors and may have caused insignificant results of true tissue changes. For example, tissue changes for MC2 were increased compared to MC0 but not significantly. What is more, limited sample number did not allow for covariate analysis. Therefore, the correlation of connective tissue (MT) with LBP (VAS back) should be taken with care. Controlling for demographic factors, psychological factors, comorbidities, and medication in larger studies are imperative to make conclusive histomorphometric statements for MC. In addition, clinical relevance of histological findings could be investigated by following up study participants.

The surgical population investigated in this study may not represent the typical MC patient because most chronic LBP patients do not undergo surgery. Surgical indication for all included patients were stenosis, deformity, instability, or a combination of them. MC was never a surgical indication. Therefore, our MC study population is likely skewed towards long lasting chronic LBP, which do not respond to conservative treatment and towards patients with pathologies that could benefit from spinal fusion. Lastly, with focusing on fibrosis-related parameters, we introduced a bias in PCA and hierarchical clustering that may result in overestimation of the relevance of fibrosis in MC. Unbiased screening approaches are required to identify novel aspect of MC.

In conclusion, this is the largest and first semi-quantitative study on MC biopsies with control samples. We found that increased numbers of CD90^+^ BMSCs are a major characteristic of MC bone marrow and associated with inflammatory and fibrotic changes in MC patients undergoing lumbar spinal fusion. Therefore, CD90^+^ BMSC may play an important role in MC pathophysiology in this study population. Targeting CD90^+^ cells could be an interesting novel treatment target for MC.

## Author contribution

(1) Conception and design, (2) Acquisition of data, analysis, or interpretation of the data, (3) Drafting of the article, (4) Critical revision of the article for important intellectual content, (5) Final approval of the article, (6) Provision of study materials or patients, (7) Statistical expertise, (8) Obtaining of funding, (9) Administrative, technical, or logistic support, (10) Collection and assembly of data.

SD: 1, 2, 3, 5, 7, 8, 10; AK: 1, 2, 4, 5, 9, 10; LG: 2, 4, 5, 9, 10; IH: 2, 4, 5, 9, 10; CL: 2, 4, 5, 10; CS: 2, 4, 5, 10; FW: 2, 4, 5, 10; MB: 2, 4, 5, 10; CG: 2, 4, 5, 10; NFA: 2, 4, 5, 10; FB: 4, 5, 8, 9; OD: 4, 5, 8, 9; MF: 4, 5, 6, 8, 9.

SD takes responsibility for the integrity of the work as a whole, from inception to finished article.

## Role of the funding source

This work was supported by the Balgrist Foundation, the VELUX Foundation, the 10.13039/501100007252Baugarten Foundation, and the Clinical Research Priority Program of the University of Zurich (CRPP Pain). Research was supported by the 10.13039/100000069National Institute of Arthritis and Musculoskeletal and Skin Diseases of the National Institutes of Health under Award Number U19AR076737and the Clinical Research Priority Program of the 10.13039/501100006447University of Zurich (CRPP Pain). The content is solely the responsibility of the authors and does not necessarily represent the official views of the 10.13039/100000009National Institutes of Health. All funding sources had no involvement in the study design, sample and data collection, analysis and interpretation of data; in writing of the manuscript; and in the decision to submit the manuscript for publication.

## Declaration of competing interest

None of the authors have a conflict of interest.
