# Supplementary material for: CD90-positive stromal cells associate with inflammatory and fibrotic changes in modic changes
Source: Osteoarthr Cartil Open. 2022 Jun 22;4(3):100287. doi: 10.1016/j.ocarto.2022.100287 (PMC9718347; doi:10.1016/j.ocarto.2022.100287)
Supplement: Multimedia component 1 [file mmc1.docx]

# **Supplementary Data 1:** MRI scanner information

All patients were scanned either with a 1.5T or 3.0T Siemens scanner. The acquisition parameters were:

|  | **Sequence** | **Plane** | **TR/TE [ms]** | **FOV [mm]** | **Slice thickness [mm]** | **Matrix** |
| --- | --- | --- | --- | --- | --- | --- |
| **1.5 Tesla** | **T2w Dixon (IP/W) TSE** | sag | 3850 / 92 | 300 x 300 | 4 | 512 x 358 |
|  | **T1w TSE** | sag | 572 / 9.1 | 300 x 300 | 4 | 512 x 358 |
|  | **T2w TSE** | ax | 4140 / 108 | 220 x 220 | 4 | 384 x 288 |
| **3 Tesla** | **T2w Dixon (IP/W) TSE** | sag | 3500 / 86 | 300 x 300 | 4 | 512 x 358 |
|  | **T1w TSE** | sag | 650 / 9.9 | 300 x 300 | 4 | 512 x 358 |
|  | **T2w TSE** | ax | 3800 / 96 | 220 x 220 | 4 | 384 x 346 |

FOV, field of view; IP, in-phase image; TE, echo time; TR, repetition time; TSE, turbo spin echo; W, water-only image.
