# Supplementary material for: CD90-positive stromal cells associate with inflammatory and fibrotic changes in modic changes
Source: Osteoarthr Cartil Open. 2022 Jun 22;4(3):100287. doi: 10.1016/j.ocarto.2022.100287 (PMC9718347; doi:10.1016/j.ocarto.2022.100287)
Supplement: Multimedia component 2 [file mmc2.docx]

# **Supplementary Data 2:** Antibodies for immunohistochemistry

| **Epitope** | **clone** | **species** | **Manufacturer** | **Order no.** | **dilution** | **detection** |
| --- | --- | --- | --- | --- | --- | --- |
| CD90 | 5E10 | Mouse | Biolegend, San Diego, CA, USA | 328101 | 1:200 | HRP |
| CD105 | SN6h | Mouse | Dako, Basel, Switzerland | M3527 | 1:100 | HRP |
| Fibronectin | IST-9 | Mouse | Santa Cruz, Dallas, TX, USA | sc-59826 | 1:200 | HRP |
| Collagen I | 3G3 | Mouse | Abcam, Cambridge, UK | ab88147 | 1:250 | HRP |
| Collagen III | polyclonal | Rabbit | Abcam, Cambridge, UK | ab7778 | 1:200 | HRP |
| aSMA | 1A4 | Mouse | Sigma Aldrich, Buchs, Switzerland | A2547 | 1:750 |  |
| anti-mouse | polyclonal | Rabbit | Dako Agilent, Santa Clara, CA, USA | D0486 | 1:50 | AP |
