# Supplementary material for: CD90-positive stromal cells associate with inflammatory and fibrotic changes in modic changes
Source: Osteoarthr Cartil Open. 2022 Jun 22;4(3):100287. doi: 10.1016/j.ocarto.2022.100287 (PMC9718347; doi:10.1016/j.ocarto.2022.100287)
Supplement: Multimedia component 3 [file mmc3.docx]

# **Supplementary Data 3:** Histological scoring system

Part A. Histopathological findings: inflammation, edema, MT

Final score = score sum I + II

Part B. Immunohistochemistry: FN, COL1, COL3, CD90, CD105

Final score= score sum (A+B), whereas 0-2 is considered negative reaction, 3-8 positive reaction

Part C. Immunohistochemistry: aSMA (expression grade in myofibroblasts/activated fibroblasts)

**Inter-class correlation of two independent single readouts of two pathologists**

**ICC3 (two-way mixed, single measures, consistency)**

| Read-out | ICC3 |
| --- | --- |
| Cellularity | 0.559 |
| Homogeneity | 0.832 |
| Inflammatory infiltrates | 0.357 |
| Oedema | 0.555 |
| Masson Trichrome | 0.678 |
| SMA | 0.201 |
| Type I collagen | 0.500 |
| Type III collagen | 0.667 |
| Fibronectin | 0.821 |
| CD105 | 0.701 |
| CD90 | 0.907 |
