# Supplementary material for: CD90-positive stromal cells associate with inflammatory and fibrotic changes in modic changes
Source: Osteoarthr Cartil Open. 2022 Jun 22;4(3):100287. doi: 10.1016/j.ocarto.2022.100287 (PMC9718347; doi:10.1016/j.ocarto.2022.100287)

**Supplementary Data 4.** Silhouette plot of k-means clustering. Using two clusters results in maximal silhouette width.


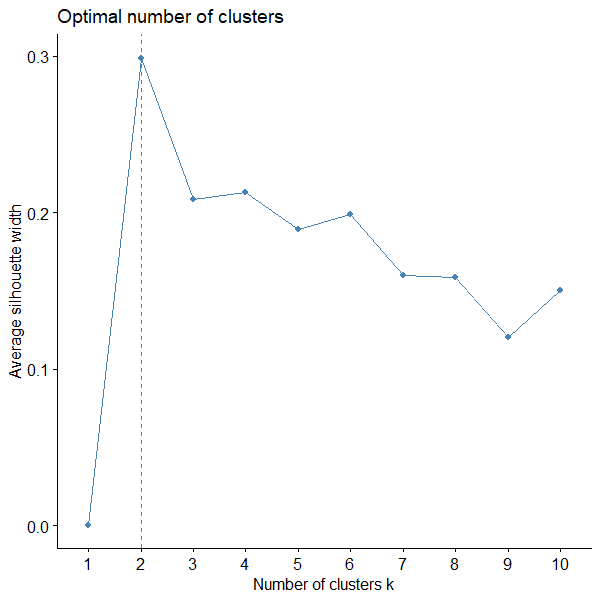

Supplement: Multimedia component 4 [file mmc4.docx]
