# Supplementary material for: CD90-positive stromal cells associate with inflammatory and fibrotic changes in modic changes
Source: Osteoarthr Cartil Open. 2022 Jun 22;4(3):100287. doi: 10.1016/j.ocarto.2022.100287 (PMC9718347; doi:10.1016/j.ocarto.2022.100287)

**Supplementary Data 5.** Comparison of Modic change biopsies of cluster 1 (n=6; 5 MC1 + 1 MC2) and Modic change biopsies of cluster 2 (n=8; 3 MC1 + 5 MC2). Data are shown as mean values ± standard deviation, except for male sex, smoker, and tissue heterogeneity, where number of individuals are reported. Cluster 1 and cluster 2 were compared with t-tests for continuous variables and with Wilcoxon test for interval data. Fisher exact tests were performed for count data. Abbreviations: MC: Modic change, MC1: Modic type 1 change, MC2; Modic type 2 change, VAS.back and VAS.leg: Visual Analogue Score for back and leg pain, respectively. ODI: Oswestry Disability Index, disc degeneration (DD), endplate scores (EPC). MT: Masson-trichrome. αSMA: alpha smooth muscle actin. COL1: type I collagen. COL3: type III collagen. FN: cellular fibronectin. CD105: endoglin. CD90: Thy-1.

|  | cluster 1 | cluster 2 (MC only) | p-value |
| --- | --- | --- | --- |
| height | 165.83 ± 14.76 | 176.63 ± 9.91 | 0.159 |
| weight | 77.92 ± 27.92 | 93.33 ± 15.77 | 0.262 |
| smoker (y/n) | 5/1 | 6/2 | 1.000 |
| VAS.back | 6.75 ± 1.71 | 6.00 ± 1.85 | 0.491 |
| VAS.leg | 6.75 ± 1.71 | 5.75 ± 3.15 | 0.797 |
| ODI | 38 ± 15.75 | 41.50 ± 14.17 | 0.668 |
| age | 59.83 ± 7.44 | 61.25 ± 13.74 | 1.000 |
| sex | 3/3 | 7/1 | 0.245 |
| DD | 4.92 ± 0.20 | 4.75 ± 0.38 | 0.416 |
| EPS | 5.75 ± 0.42 | 4.94 ± 1.15 | 0.189 |
| cell | 0.58 ± 0.20 | 1.81 ± 0.53 | **0.004** |
| homo/hetero | 1/5 | 2/6 | 1.000 |
| infla | 2.33 ± 1.51 | 0.50 ± 0.96 | **0.026** |
| oedema | 1.83 ± 0.41 | 0.38 ± 0.52 | **0.003** |
| MT | 3.00 ± 0.77 | 1.13 ± 1.13 | **0.011** |
| SMA | 1.08 ± 0.80 | 1.00 ± 0.27 | 1.000 |
| COL1 | 4.17 ± 1.72 | 3.00 ± 0.76 | 0.114 |
| COL3 | 5.83 ± 1.13 | 5.63 ± 1.19 | 0.744 |
| FN | 5.58 ± 1.39 | 3.19 ± 1.65 | **0.021** |
| CD105 | 4.54 ± 1.68 | 1.34 ± 1.25 | 0.104 |
| CD90 | 4.54 ± 1.68 | 1.34 ± 1.25 | **0.004** |


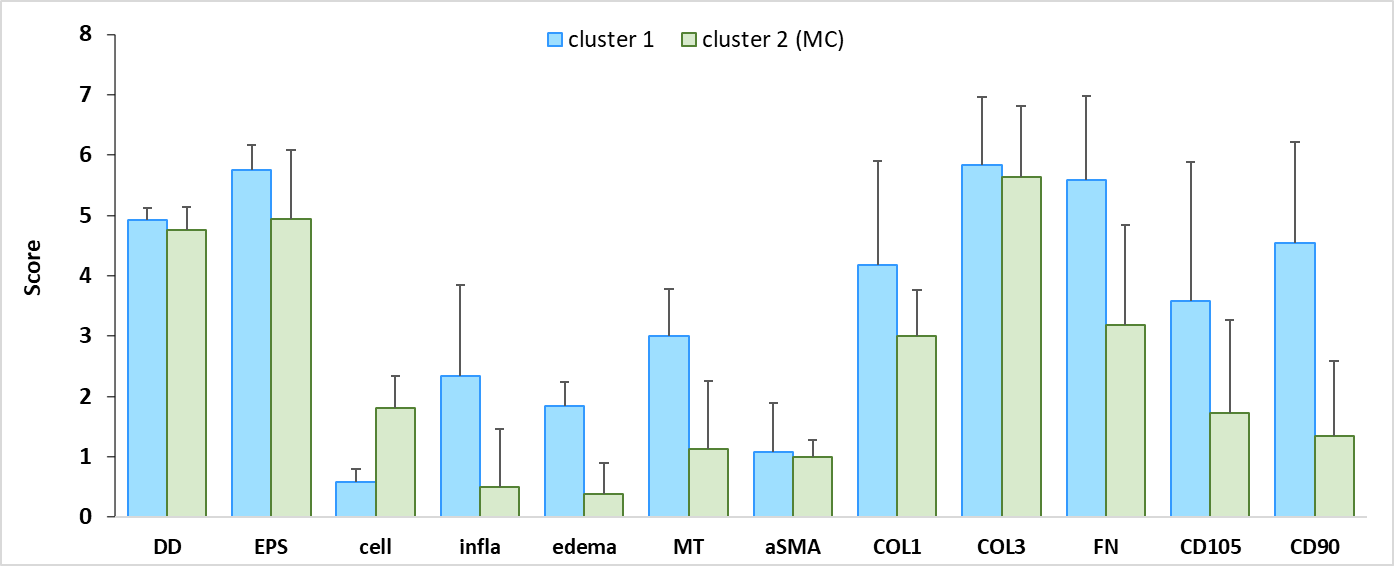

Supplement: Multimedia component 5 [file mmc5.docx]
